# From Pericarditis to Leukemia: A Rare t(4;22)(q12;q11.2) Translocation in Chronic Myeloproliferative Disorder

**DOI:** 10.51894/001c.144416

**Published:** 2025-09-30

**Authors:** Albert Alvarado

**Affiliations:** 1 Department of Internal Medicine Henry Ford Genesys

## 62

### INTRODUCTION

Chronic Myeloid Leukemia (CML) is predominantly driven by the Philadelphia chromosome arising from t(9;22)(q34;q11), which generates the BCR-ABL1 fusion gene and initiates constitutive tyrosine kinase activity. The development of tyrosine kinase inhibitors (TKIs) has markedly improved outcomes for patients with classical CML. However, atypical chromosomal aberrations and rare translocations can yield CML-like phenotypes yet differ in clinical course and therapeutic responsiveness.

### CASE PRESENTATION

A 37-year-old female presented with chest discomfort initially suspected as pericarditis, which resolved with NSAIDs. Incidental laboratory findings revealed marked leukocytosis (WBC 109.6 × 10^9/L), normocytic anemia, and a left-shifted granulocytic series. Bone marrow biopsy demonstrated hypercellularity, 3% blasts, and increased eosinophils without fibrosis. Flow cytometry identified a minor population of aberrant myeloid blasts, suggestive of a myeloproliferative disorder. Cytogenetic and molecular studies ruled out the classic BCR-ABL1 fusion but uncovered a rare t(4;22)(q12;q11.2) translocation associated with PDGFRA rearrangement.

### DISCUSSION

Given the absence of BCR-ABL1, definitive guidelines for therapy were not straightforward. Nevertheless, due to the high leukocyte count and eosinophilia, the patient was initiated on hydroxyurea for cytoreduction and corticosteroids to control eosinophilic activity, with plans to transition to imatinib. Although TKIs are primarily designed to target BCR-ABL1, some agents may offer partial benefit in PDGFRA-related disorders. Hematopoietic stem cell transplantation was also recommended because of the complex karyotype and limited data on long-term efficacy of TKIs in such rare translocations.

### CONCLUSION

This case underscores the importance of comprehensive genetic and molecular evaluations in hematologic malignancies, especially when clinical features mimic CML yet lack the canonical BCR-ABL1 fusion. The discovery of the t(4;22)(q12;q11.2) translocation highlights the genetic heterogeneity within myeloproliferative neoplasms and necessitates individualized diagnostic and treatment strategies. Ongoing research into these atypical rearrangements is crucial for elucidating their molecular mechanisms and developing more targeted therapies. Ultimately, a personalized approach that integrates cytogenetic, molecular, and clinical data offers the greatest promise for improving outcomes in patients with rare and genetically complex myeloproliferative disorders. Continued investigation into novel targeted agents will further refine management strategies and potentially enhance long-term survival.

